# Review of WADA Prohibited Substances: Limited Evidence for Performance-Enhancing Effects

**DOI:** 10.1007/s40279-018-1014-1

**Published:** 2018-11-08

**Authors:** Jules A. A. C. Heuberger, Adam F. Cohen

**Affiliations:** 1 Centre for Human Drug Research, Zernikedreef 8, 2333 CL Leiden, The Netherlands; 2 Department of Internal Medicine, Leiden University Medical Centre, Leiden, The Netherlands

## Abstract

The World Anti-Doping Agency is responsible for maintaining a Prohibited List that describes the use of substances and methods that are prohibited for athletes. The list currently contains 23 substance classes, and an important reason for the existence of this list is to prevent unfair competition due to pharmacologically enhanced performance. The aim of this review was to give an overview of the available evidence for performance enhancement of these substance classes. We searched the scientific literature through PubMed for studies and reviews evaluating the effects of substance classes on performance. Findings from double-blind, randomized controlled trials were considered as evidence for (the absence of) effects if they were performed in trained subjects measuring relevant performance outcomes. Only 5 of 23 substance classes show evidence of having the ability to enhance actual sports performance, i.e. anabolic agents, β2-agonists, stimulants, glucocorticoids and β-blockers. One additional class, growth hormone, has similar evidence but only in untrained subjects. The observed effects all relate to strength or sprint performance (and accuracy for β-blockers); there are no studies showing positive effects on reliable markers of endurance performance. For 11 classes, no well-designed studies are available, and, for the remaining six classes, there is evidence of an absence of a positive effect. In conclusion, for the majority of substance classes, no convincing evidence for performance enhancement is available, while, for the remaining classes, the evidence is based on a total of only 266 subjects from 11 studies.

## Key Points

**Table:** 

This review shows that only 5 of 23 substance classes on the World Anti-Doping Agency Prohibited List show robust evidence of having the ability to enhance actual sports performance in athletes.
A total of 11 studies including 266 subjects form all the available level 1 evidence for positive pharmacological effects on strength and sprint performance; there are no studies showing benefit on endurance performance.

## Introduction

The mission of the World Anti-Doping Agency (WADA) is to lead a collaborative worldwide movement for doping-free sport, and its activities focus on the responsibilities given by the World Anti-Doping Code [1]. One of these responsibilities is to publish an annual Prohibited List, which identifies the substances and methods prohibited in- and out-of-competition, and in particular sports [2]. This list is compiled by the List Expert Group and Health, Medical and Research Committee of the WADA, in consultation with scientific, medical and anti-doping experts, using criteria described in the World Anti-Doping Code. This describes that a substance or method shall be considered to be placed on the Prohibited List if the substance or method meets any two of the following three criteria [1].

1. Medical or other scientific evidence, pharmacological effect or experience that the substance or method, alone or in combination with other substances or methods, has the potential to enhance, or enhances, sport performance.
2. Medical or other scientific evidence, pharmacological effect or experience that the use of the substance or method represents an actual or potential health risk to the athlete.
3. WADA’s determination that the use of the substance or method violates the spirit of sport, described in the introduction to the World Anti-Doping Code.

The third criterion is clearly most subjective and is more a fundamental and philosophical question than a scientific one [3]. However, the remaining two criteria do mention the availability of scientific evidence, indicating that the decision for placing substances and methods on the Prohibited List could be evidence-based. So how strong is this evidence for the listed substances? In this review, we specifically focus on the evidence for performance enhancement, although there could be other reasons athletes use prohibited substances, including masking or diminishing the side effects of other prohibited substances. Several reviews are available focusing on the performance effects of different categories on the Prohibited List [4–7]; however, the current review aims to provide a comprehensive and up-to-date overview of the evidence for performance enhancement of all categories of substances in- and out-of-competition on the 2018 Prohibited List, applying standards considered appropriate in clinical therapeutics.

## Methodological Considerations

The 2018 Prohibited List was used as framework for this review [2]. We searched the scientific literature for studies and reviews evaluating the clinical effects of the different substances and categories of substances on performance using PubMed as the search engine. Scientific articles with no date restriction and with combinations of the following keywords were evaluated for their relevance by title and abstract: ‘athletes’, ‘performance’, ‘sport’, ‘doping’ and ‘trained’, in combination with a specific prohibited compound or category (e.g. ‘terbutaline’ or ‘β2-agonist’). Reference lists of identified publications were searched for additional relevant publications. Performance was interpreted according to the broadest sports-related definition, including strength (power) and endurance. Although the criterion in the WADA Code states that evidence for *the potential* to enhance performance is sufficient to place a substance on the Prohibited List, in this review clinical pharmacological evidence for actual performance enhancement was considered essential to determine that a substance or category of substances has a positive effect on performance. In other words, similar to any other therapeutic review, to make an evidence-based conclusion that there are performance-enhancing effects, the level of evidence should preferably be high (level 1), meaning that evidence should come from double-blind, randomized controlled trials (or meta-analysis based on randomized controlled trials) [8]. This was also taken into account when evaluating the search results, although there will inevitably be cases where information has to be inferred from other, less-reliable evidence. In addition, ideally these trials should measure relevant performance outcomes, and therefore we defined which outcomes should be considered most relevant. In this review, we apply the same standard as for clinical trials, where proven effects on clinical outcome are accepted as most reliable, and effects on surrogate markers that have a proven link to that clinical outcome are accepted, as, for example, described by the US FDA [9]. When translated to sport performance, the most relevant outcome measure is the ‘actual’ performance of the sport itself, such as, for example, muscle strength for weight lifting, or running time for distance running. However, surrogate markers that describe an important aspect of the performance might be acceptable, but conclusions based on such markers can only be reliable if there is a proven high correlation with the actual performance. For endurance performance, for example, although maximal oxygen consumption (*V*O_2max_) is often used and has been shown to be a prerequisite for performance [10, 11], its predictive value for endurance performance within a group of athletes is very limited [12, 13]. Moreover, it seems that successful endurance athletes reach a plateau in *V*O_2max_ despite continuing to improve performance [14–16], thereby questioning whether increasing *V*O_2max_ by any means would have an impact on performance, at least in highly trained subjects. Finally, there has been critique on the use of the maximal exercise test that generates the *V*O_2max_ marker, to accurately evaluate athletic potential in general as it does not resemble normal exercise [17]. It is therefore unclear whether a pharmacological effect on *V*O_2max_ (or other maximal exercise test markers) translates into an effect on performance per se, making it a marker with insufficient predictive value. Another test that is not very reliable in measuring effects on actual performance is the time-to-exhaustion test. Such a test has been shown to have low reproducibility, especially compared with time trials that continue for a predetermined amount of time or work [18, 19]. Moreover, there is no clear evidence of their correlation with actual performance, except for the absence of a correlation with Ironman performance in one study [20]. This is possibly because sports disciplines do not rely on time-to-exhaustion principles, but rather on pacing to a finish line or time. In summary, there currently are no widely recognised laboratory markers for (aerobic) endurance performance, leaving tests for actual endurance performance (e.g. a time trial) as the most reliable available measure. On the other hand, markers for sprint performance, for example as measured by a Wingate test, do resemble actual performance, such as sprinting in cycling, and this surrogate marker has also been shown to correlate with other performance types [21, 22], which is why we considered it to be a relevant marker.

Finally, the training status of study participants is a relevant factor when interpreting the outcome. The aim of preventing performance advantages through doping, as described in the WADA Code, is most (although admittedly not solely) relevant in high-level (and in particular professional) sports due to the attention, fame and commercial considerations involved in that level of sports. Clinical studies should reflect the ‘target population’, which in this case would be elite and professional athletes. However, because of doping/WADA regulations, it was/is very challenging, or even impossible, to conduct intervention studies of banned substances in such a population. For this reason, we considered studies in (highly) trained athletes most relevant, so that observed effects apply to this level of athletes, and that extrapolation of observed effects in this population to the performance of professional athletes was most valid. However, data in less well-trained subjects may also be of value, and was also reviewed. Determining the training level of subjects was based on commonly used markers for performance where possible. For the level of training in endurance performance, *V*O_2max_ and maximal power output (*P*_max_) were used. Three categories were defined somewhat arbitrarily (without taking the type of maximal exercise testing protocol into consideration): untrained (*V*O_2max_ < 55 ml/min/kg and/or *P*_max_ < 3.5 W/kg); trained (*V*O_2max_ ≥ 55 and < 65 ml/min/kg and/or *P*_max_ ≥ 3.5 and < 5.0 W/kg); and highly trained (*V*O_2max_ ≥ 65 ml/min/kg and/or *P*_max_ ≥ 5.0 W/kg). For strength training, it was more difficult to objectively categorize study populations as available measurements varied widely between included studies. Therefore, subjects were categorized as trained or untrained based on the description in the article of whether subjects had been actively engaged in resistance training.

## Findings

### Prohibited at All Times

#### S0: Non-approved Substances

Any pharmacological substance that has no current approval by any governmental regulatory health authority for human therapeutic use belongs in this category, making the category very broad. Substances in this category could be drugs under preclinical or clinical development, discontinued drugs, designer drugs, or substances approved for veterinary use only. In any case, they will be substances that (currently) lack solid evidence for (beneficial) effects in humans in general, and therefore, in practically all cases, lack evidence for enhancement of performance in particular.

#### S1: Anabolic Agents

Anabolic agents, or anabolic-androgenic steroids (AAS), are synthetic derivatives of testosterone that have attracted attention as doping substances due to their potential to increase protein synthesis and decrease protein breakdown (anabolic effects) and increase muscle growth (androgenic effects) by activating the androgen receptor. In 2004, a very thorough review evaluated the evidence for the effects of AAS on performance [23].

Upon inspection of the studies covered in this current review, there were various studies with a randomized, double-blind, controlled design that investigated the effects on strength. The most recent of these studies shows clear effects of AAS on different strength outcomes in strength-trained men, alone and combined with strength training [24–26]. One of these studies showed, with an elegant design, that high-dose testosterone (600 mg/week), both with and without strength training, significantly increased bench-press and squatting power by, on average, 10–20% compared with the respective placebo condition [24]. Other, well-designed studies covered in the review, investigating the effects on endurance performance, or related measures such as *V*O_2max_, are older and more sparse. These showed no treatment-induced improvements, although they did not show an effect on strength either, indicating the sample size or dose might be too small to detect effects [27, 28]. However, since the review, an additional randomized, placebo-controlled trial has been conducted that also showed a lack of effect on endurance performance markers after 1 month of AAS in doses similar to those that showed strength effects [29]. Additionally, this study showed evidence that there is no effect of AAS treatment on recovery. In summary, high-dose AAS appears to increase strength but not endurance performance. The evidence on strength effects is based on three studies including a total of 91 volunteers.

#### S2: Peptide Hormones, Growth Factors, Related Substances and Mimetics

##### Erythropoietins and Agents Affecting Erythropoiesis

These agents are aimed at increasing red blood cell volume through inducing erythropoiesis, and thereby potentially enhancing performance. Interestingly, for ‘natural’ increases in red blood cell volume through altitude training, the evidence for performance-enhancing effects is not fully convincing [30].

*Erythropoietin-Receptor Agonists* Erythropoietin-receptor agonists, such as recombinant human erythropoietins (rHuEPOs), stimulate erythropoiesis and thereby increase haemoglobin levels, which potentially increases oxygen carrying capacity and hence improves endurance performance. However, a systematic review of the literature by Heuberger et al. concluded that there was a lack of evidence for efficacy on endurance performance [31]. Of the 13 available reviewed studies, only 5 had a placebo-controlled and double-blind design [32–36], all showing similar effects of rHuEPOs in both trained and untrained subjects. In all studies, *V*O_2max_ increased by approximately 7%, while *P*_max_, which was evaluated in two of the studies, also increased by 7% [33, 36]. Finally, time to exhaustion improved by 22% in untrained subjects [33] and 9.4% in trained subjects [32]. Two subsequent randomized, placebo-controlled trials also showed increases in *V*O_2max_, *P*_max_ and time to exhaustion of 5%, 6% and 58%, respectively, in trained subjects [37], and an increase in *V*O_2max_ of 6%, but no increase in time to exhaustion, in untrained subjects [38]. However, none of these studies showed whether these effects on surrogate biomarkers impacted actual performance. Because of this lack of information, a double-blind, randomized, placebo-controlled study in trained cyclists followed and showed that clinically more relevant tests, such as a time trial and uphill road race, were not affected by rHuEPO treatment [39]. Although there was again an effect of rHuEPOs on maximal exercise test variables, including *V*O_2max_ and *P*_max_ (an increase of 5 and 3%, respectively), there was no evidence to suggest that these erythropoietin-induced effects improved actual cycling performance in trained cyclists. The absence of an effect on these measures most related to competitive (cycling) performance in athletes is insightful, but one should be cautious about extrapolating these findings to all performance types in elite athletes—not all performance aspects of endurance have been studied, and the target population has not been included. In any case, there is no evidence showing that rHuEPOs enhance time trials, climbing or other race performance in athletes.

*Hypoxia-Inducible Factor Activating Agents* Hypoxia-inducible factor (HIF) activating agents have a direct effect on erythropoietin production by stimulating erythropoietin gene expression, therefore the same rationale for potential performance-enhancing effects applies as for direct rHuEPO administration. As shown for rHuEPOs in the section above, increases in erythropoietin, and the accompanying increases in haemoglobin, have not been shown to improve endurance performance in trained subjects. Moreover, evidence for the effects of HIF activating agents is even more sparse. Cobalt has been observed to increase erythropoiesis in anaemic patients [40, 41]. No trials have been performed evaluating these effects on erythropoiesis or performance in healthy volunteers, let alone athletes, as can be seen in the review by Ebert and Jelkmann [42]. More recently, a study claimed to show the effects of xenon on erythropoietin production in healthy volunteers [43], but the statistics of the study have been criticized [44]. Small molecule HIFs are in clinical development but have not yet been approved for clinical use. The published clinical studies show that these compounds produce modest increases in erythropoietin in both anaemic patients and healthy volunteers [45, 46], however there are currently no studies evaluating effects on the performance of healthy or trained subjects.

*GATA Inhibitors* By inhibiting GATA, an erythropoietin gene expression inhibitor, a similar effect as for the HIF activating agents could be expected; however, there are no published clinical studies on the effects of these compounds—the mechanism has only been proven preclinically [47, 48].

*Transforming Growth Factor-β Inhibitors* Erythropoietin induction by transforming growth factor (TGF)-β inhibition is a very recent development in the possible treatment of anaemia, and in particular for myelodysplastic syndromes. Luspatercept and sotatercept have been shown to increase haemoglobin levels in such patients [49, 50], but there is no evidence regarding any related effects on performance in healthy or trained individuals.

*Innate Repair Receptor Agonists* Innate repair receptor agonists are non-erythropoietic derivatives of rHuEPO that have been developed for their potential tissue-protective properties, and that to date have only been evaluated in a few clinical trials. One published placebo-controlled trial indicated carbamylated erythropoietin was safe and well-tolerated [51], but no evidence of performance effects is available.

##### Peptide Hormones and Hormone Modulators

*Chorionic Gonadotrophin and Luteinizing Hormone and Their Releasing Factors* Chorionic gonadotrophin (CG) and luteinizing hormone (LH) are hormones that bind to the same receptor (LHCG receptor), which has several functions in the reproductive system. In females, follicular maturation, ovulation and luteal function are influenced through stimulation of the receptor in the ovary, while in males the receptor is located in the testis and stimulates testosterone production. There is no indication that the effects in females can positively influence performance [52], but the increase in testosterone in males may give similar effects as those described for anabolic agents. For example, a single intramuscular injection of 6000 IU of CG increased testosterone levels by approximately 40 nmol/l in healthy men [53]. This is half the increase observed after a 10-week treatment with 600 mg of testosterone enanthate (an anabolic steroid), which has been shown to increase bench press and squat muscle strength [24]. However, no studies have investigated the effects of GC or LH on any sports performance measures.

*Corticotrophins and Their Releasing Factors* Adrenocorticotropic hormone (ACTH) is involved in the hypothalamic-pituitary-adrenal axis and is released in response to stress, leading to increases in cortisol. Through this cortisol response, free fatty acids are released, potentially sparing glycogen, which is then assumed to benefit endurance performance. In addition, ACTH stimulates glucocorticoid secretion (see Sect. 3.2.4). However, a double-blind, placebo-controlled, crossover study in 16 trained cyclists showed that although a 1 mg ACTH depot dose decreased the feeling of fatigue during a submaximal effort, it did not improve maximal performance in a maximal exercise test, nor did it affect recovery between two consecutive tests [54]. Similarly, 20-km time-trial performance was not affected by 0.25 mg ACTH intramuscular injections in a double-blind, placebo-controlled, crossover study in eight (highly) trained male cyclists [55]. Perceived fatigue was not decreased by ACTH in this study. As these are the only studies performed, we conclude there is no evidence of beneficial effects of ACTH or its releasing factors on actual performance.

*Growth Hormone, Its Fragments and Releasing Factors* Growth hormone (GH) use in adults with GH deficiency results in reduced body fat, increased lean body mass and increased fitness and strength [56], and has therefore attracted attention as a potential performance-enhancing drug. This mechanism is mainly mediated by insulin-like growth factor (IGF)-1. A systematic review evaluated the effects on strength or endurance performance [57]. For strength, two double-blind studies were identified that showed no effects of GH on the muscle strength of different muscles compared with placebo when combined with strength training in untrained [58] and trained strength athletes [59]. Endurance performance was evaluated in two double-blind studies; multiple dosing of GH did not have an effect on *V*O_2max_ and *P*_max_ compared with placebo in trained subjects [60]. In a crossover design, a single dose of GH increased plasma lactate levels during submaximal cycling exercise compared with placebo in seven highly trained cyclists [61]. Such single administrations of GH therefore seem to decrease endurance performance, underlined by the fact that three of seven cyclists in this study had difficulty completing the cycling trial when treated with GH, compared with none receiving placebo treatment. Following the review, one randomized, placebo-controlled, blinded trial with 8 weeks of daily GH treatment confirmed these findings and showed no effects on strength or *V*O_2max_. In this study, there was however an increase in sprint performance in a 30 s maximal sprint test (Wingate test) of approximately 1 kJ (or a 3.9% relative increase in the combined male and female group, and a 5.5% relative increase for the male group only), which was slightly larger when GH was coadministered with weekly testosterone doses [62]. It should be noted that GH also increased the incidence of swelling, joint pain and paraesthesia in this study, indicating these gains are not without downsides and possible risks. Additionally, subjects were untrained for endurance, therefore it is difficult to know how this effect on sprint performance extrapolates to elite athletes.

##### Growth Factors and Growth Factor Modulators

Blood platelets can release growth factors for example when triggered by signs of injury. These could potentially be used for treating sports injuries [63], but are also thought to provide benefit in healthy athletes. However, for most of these factors, including fibroblast, hepatocyte, mechano, platelet-derived and vascular endothelial growth factors, and thymosin-β4, there are no studies regarding the effects on performance. The only available studies evaluated the safety or efficacy of these products in healthy volunteers and patients [64–66]. However, there is one exception: IGF-1, which is thought to possess ergogenic effects mainly through the anabolic pathway that is shared with GH, has been investigated as an ergogenic aid. A randomized, double-blind, placebo-controlled study investigated the effects of a recombinant human IGF-1/IGF-binding protein-3 complex (rhIGF-I/rhIGFBP-3) on body composition and aerobic performance in untrained persons [67]. No effects on body composition were observed but an increase in *V*O_2max_ was reported for both low (30 mg/day) and high (60 mg/day) doses. However, the conclusion that IGF-1 therefore improves aerobic fitness should be interpreted with caution. First, changes in outcome parameters were only analysed within each group and were not compared with the placebo group, which would have been the appropriate analysis in such a study design. Second, even if the observed effect on *V*O_2max_ is truly caused by IGF-1, it is unclear if this has an impact on actual performance. Unfortunately, no performance parameter such as running speed on the treadmill test was reported, nor was a test performed mimicking actual sports performance. In addition to the fact that participants were untrained, this makes it impossible to interpret what these findings mean for the performance of elite athletes.

#### S3: β2-Agonists

β2-Agonists are used in the treatment of asthma as they act as bronchodilators through their relaxing effect on the smooth muscles of the lung via the β2-adrenergic receptor. In addition, they have an effect on muscle tissue through this pathway, and both actions have been implied to possess performance-enhancing effects. Several extensive reviews have evaluated the evidence for this. In a 2011 systematic review based on a meta-analysis of randomized controlled trials, Pluim et al. [68] concluded that there are no positive effects of inhaled β2-agonists on endurance, strength or sprint performance, and that there was insufficient evidence to draw conclusions regarding systemic β2-agonist use. In 2015, Cairns and Borrani [69] had more systemic dosing studies at their disposal, and concluded in their review that only high-dose systemic β2-agonists (at a serum concentration of approximately 0.1 μmol/l) have a positive effect on muscle strength and peak sprint power. This is based on the observation, in a placebo-controlled, randomized, crossover design in highly trained and trained men, that after oral administration of 20–25 mg terbutaline, sarcoplasmic reticulum rates of Ca^2+^ release and uptake were increased, together with maximal voluntary isometric contraction (+ 6%) and peak twitch force (+ 11%) [70]. No effects on time to exhaustion were observed. High-dose (15 mg) inhaled terbutaline reached similar serum concentrations (approximately 0.1 μmol/l) in another double-blind, randomized, crossover trial, and increased quadriceps muscle strength by 8.4%. In addition, Wingate peak and mean power increased by 2.2% and 3.3%, respectively, and Wingate total work increased by 3% compared with placebo in trained males, but time-trial performance was not affected [71]. A double-blind, randomized, placebo-controlled study in highly trained athletes published after the review by Cairns and Borrani [69] showed that single and 2-week dosing of 8 mg salbutamol had no effect on body mass, *V*O_2max_, incremental peak power output, time to exhaustion, maximal voluntary isometric contraction or isometric endurance. However, there was a significant increase in Wingate peak power of 4% and 6% for single and multiple dosing, respectively, similar to the inhaled terbutaline study [72]. The statistical analysis in this study did not include a comparison to the placebo treatment, but because no significant effect was observed in the placebo group, the increase in the salbutamol group seemed to be a true effect. In all three studies, subjects experienced mild side effects, namely tremor and tachycardia. Only one additional study showed the effects of inhaled administered β2-agonists. In this case, the effect was only seen in one very specific task, of which the clinical relevance is questionable, namely quadriceps endurance in highly trained endurance athletes [73], and therefore the vast majority of evidence shows no ergogenic effects of inhaled β2-agonists. Overall, these findings indicate that only high β2-agonist concentrations, which are mainly achieved by systemic administration, can improve performance, but only in strength and very short disciplines requiring high-power development, as represented by the Wingate test, and at the cost of tremor and tachycardia. This evidence is based on three studies with a total of 39 volunteers.

#### S4: Hormone and Metabolic Modulators

##### Aromatase Inhibitors

Aromatase inhibitors lead to reduced enzyme activity for the conversion of androgens to oestrogens. This in turn leads to lowered oestrogen levels, and thereby, via inhibition of negative feedback on the hypothalamus, to higher testosterone levels. This increase has been shown to be approximately 15 nmol/l in healthy males, for exemestane [74]. As for CG and LH, there are no trials investigating the effects of these aromatase inhibitors on performance, and the only indication of potential effects is an increase in testosterone, which is approximately 25% that observed after AAS treatment, leading to increased muscle strength [24]. Evidence is therefore similarly weak as described for GC and LH.

##### Selective Estrogen Receptor Modulators

The evidence basis for selective estrogen receptor modulators (SERMs) is similar to that for aromatase inhibitors. SERMs, such as tamoxifen and raloxifen, are clinically used for their estrogenic and anti-estrogenic effects in different tissues. This induces increases in pituitary gonadotrophin secretion and, consequently, increases in testosterone levels in men, seemingly somewhat smaller than for aromatase inhibitors [75]. There are no studies investigating the effects of SERMs on performance.

##### Other Anti-estrogenic Substances

The examples mentioned in the 2018 Prohibited List in this category, clomiphene and cyclofenil, are older SERMs (although perhaps less selective than, for example, tamoxifen). As effects are similar to compounds described in the previous section, and there are no studies on performance enhancement [76, 77], the conclusion about the evidence for performance effects is the same—no evidence is available. Another substance in this category, fulvestrant, is a selective estrogen receptor degrader with no effects that could clearly enhance performance, and no evidence that it does so.

##### Agents Modifying Myostatin Function(s)

Myostatin is a negative regulator of muscle growth, therefore lowering its levels or inhibition of its action could potentially increase muscle size and improve performance. Although muscle growth is observed in some preclinical studies, it is questionable if this also results in increased strength, as reviewed by Fedoruk and Rupert [78]. In addition, there are currently no approved drugs (developed for diseases with muscle weakness or wasting) in this class yet [79], therefore there is presently no evidence of the effects on performance in athletes.

##### Metabolic Modulators

There are several substance types in the metabolic modulators category. Peroxisome proliferator-activated receptor (PPAR)-δ agonists and AMP-activated protein kinase (AMPK) activators might enhance performance through their effects on energy expenditure and substrate utilization. In mice, a PPAR-δ agonist, as well as an AMPK agonist [i.e. 5-aminoimidazole-4-carboxamide ribonucleotide (AICAR)], increased running endurance [80]. However, there are currently no approved PPAR-δ agonists [81], and neither is there evidence for performance enhancement in humans. Similarly, specific AMPK activators are not approved (such as AICAR), although there are approved drugs that have an AMPK activating effect, e.g. metformin, which is not prohibited. However, clinical studies evaluating the effects on performance in healthy subjects are sparse, as reviewed by Niederberger et al. [82]. That review cites two studies evaluating metformin effects in healthy volunteers, one of which was a multiple-dose, double-blind, placebo-controlled crossover trial [83]. The blinding of this study was described as not being optimal (due to taste and gastrointestinal side effects), randomization is not described, and there was no baseline measurement for each treatment, making the conclusions less robust. Nevertheless, no positive effect on performance markers was observed. Moreover, a small but significant decrease in *V*O_2max_ and time to exhaustion in the maximal test was found in the metformin treatment group. The second study was a randomized, double-blind, placebo-controlled, single-dose, crossover study that showed no difference between treatments, although this study also did not include a baseline measurement [84]. In both studies, participants were untrained.

With regard to insulin, Kuipers and van Dugteren indicated that based on several observations, this drug is not expected to have a physiologically significant effect on muscle growth, even in combination with glucose and/or amino acids [85]. However, no studies have been published assessing the effects of insulin on performance.

Finally, inhibitors of fatty acid oxidation belong to this category. Meldonium is classified as a partial inhibitor of fatty acid oxidation, but, in a recent editorial, Greenblatt and Greenblatt concluded that no studies have evaluated the performance-enhancing properties of meldonium in trained subjects [86]. Another inhibitor of free fatty acid oxidation, trimetazidine, was reported to improve maximal walking distance in patients with peripheral arterial disease [87], but there is no evidence of such an effect on exercise performance in healthy or trained individuals.

#### S5: Diuretics and Masking Agents

The category of diuretics and masking agents is not necessarily included in the Prohibited List for their potential to enhance performance. Masking agents are supposed to interfere with analytical testing of markers or other substances on the Prohibited List. Diuretics increase urine production and by this effect are thought to dilute, and therefore interfere with the detection of, banned substances in urine. This increased water excretion caused by diuretics might also improve performance as it can quickly reduce weight, which might give a competitive advantage. In sports with weight classes, for example, this effect could place athletes in a lighter category, and, in speed or endurance sports, lighter athletes might have an advantage. Cadwallander et al. [88] reviewed the effects of diuretics, but it should be noted that some of the studies were not placebo-controlled, and only used a control condition. Although it could be argued that the diuretic effect would have deblinding effects anyway, the results should be interpreted with caution. Caldwell et al. [89] showed that two doses of approximately 60 mg of furosemide decreased work load during a maximal exercise test and decreased *V*O_2max_ compared with baseline measurements, but not compared with controls, in untrained subjects. Armstrong et al. [90] found that trained runners had an impaired running time in 1500, 5000 and 10,000 m races after 40 mg of furosemide, a difference that was significant at the two longest distances versus controls. A third study did not find an effect of a 1000 mg infusion of acetazolamide on 30 s peak or average cycling power, although it did seem to decrease peak *V*O_2_ uptake during this test [91]. Another study evaluated the effects of a single dose of 500 mg acetazolamide in a quasi-randomized, double-blind, placebo-controlled, crossover study and found that there was no effect on *V*O_2max_, but time to exhaustion was reduced by 29% in a continuous exercise to exhaustion [92]. Finally, a double-blind, placebo-controlled, crossover study in untrained subjects investigated the effects of four doses of 250 mg acetazolamide every 8 h and found a decrease in *V*O_2max_ and *P*_max_ [93]. An additional study that was not covered in the review by Cadwallander et al. [88] showed that in a randomized, double-blind, placebo-controlled, crossover study, 250 mg acetazolamide three times daily for 2 days did not significantly affect knee extension maximum voluntary contraction at the beginning of the test or at exhaustion in untrained subjects [94], but did decrease endurance performance. Overall, not all study designs were sufficiently robust and most included untrained subjects, therefore definite conclusions cannot be made regarding the performance-enhancing properties of diuretics. Nonetheless, given the available studies, if anything the evidence indicates that athletic performance is negatively affected by diuretics.

#### M1-3: Prohibited Methods

There are several non-pharmacological interventions that are prohibited at all times, termed prohibited methods. These are manipulation of blood and blood components (e.g. blood transfusion), chemical and physical manipulation (e.g. tampering with a sample or intravenous infusions of fluid) and gene doping. As this review focuses on pharmacological interventions, evidence for the effects on performance of these categories is not discussed here.

### Prohibited In-Competition

#### S6: Stimulants

Stimulants are thought to potentially improve performance via the effects on neurotransmitter levels in the brain, predominantly dopamine and norepinephrine. Research into the effects of stimulants on performance has mainly focused on a few drug classes. Amphetamines such as amphetamine sulfate [95] showed positive effects on muscle strength (knee extension strength + 23%), acceleration (+ 4%) and time to exhaustion (+ 5%) in untrained subjects. Similarly, methylphenidate [96] improved time to exhaustion (+ 29%) in highly trained subjects. *V*O_2max_ was not affected in either study and endurance performance (such as a time trial) was not investigated in these studies. Of note, the former study used no baseline correction (i.e. amphetamine performance was directly compared with placebo performance in the randomized, crossover design) and, for the latter study, it is unclear whether it was (double-)blinded, which may both make the results less robust. Another study with a higher dose of methylphenidate showed no effect on time-trial performance in normal temperature, but there was an improvement of 15% average power output compared with placebo in the heat (30°) in trained subjects [97]. Levomethamphetamine was investigated for its effect on time-trial performance in young participants and showed no change [98].

Ephedrine, pseudoephedrine and phenylpropanolamine have a similar mechanism of action to amphetamines. Two studies investigating the effects of ephedrine showed positive effects. One study found an effect on peak Wingate sprint power (+ 0.6%), but not on time to exhaustion [99], in untrained subjects, and another study found an improvement in a type of time-to-exhaustion test in trained strength athletes, namely leg and bench press repetitions (+ 30% and + 8%, respectively) [100]. One positive study for pseudoephedrine used a dose of 180 mg, which increased knee extension strength by 9% and peak Wingate sprint performance by 3%, but not bench press power, in strength-trained subjects [101]. Later publications also showed that low doses of pseudoephedrine used clinically did not affect 5000 m run time in highly trained runners [102], or peak power or total work during a Wingate test in trained subjects [103]; only high doses improved performance, with 1500 m run time decreasing by 2% in highly trained runners [104]. The authors of this latter study therefore concluded that high pseudoephedrine doses are needed for performance effects.

For cocaine, another well-known stimulant that is on the Prohibited List, there are no well-designed studies evaluating its effects on performance.

Overall, studies on the effects of these stimulants show varying results, making it unclear whether they improve performance, as was concluded in a review published by Clarkson and Thompson in 1997 [6]. In certain conditions and performance tests, they may modestly improve performance if administered in sufficiently high doses, but there is insufficient conclusive evidence to determine how they affect most actual sports performance types. The available evidence consists of the results of two studies involving a total of 29 volunteers.

#### S7: Narcotics

The narcotics category consists of strong analgesics, all belonging to the opioids class. Surprisingly, although not all opioids are currently banned (e.g. tramadol is allowed), substances such as morphine and its analogs, and fentanyl and its derivatives, are. Although analgesic effects might enhance performance, common side effects of opioids, including nausea, sedation and respiratory depression, would equally argue against any beneficial effects. One study showed that an intrathecal injection of fentanyl did not impact average power output during a 5-km cycling time trial in trained cyclists [105]; however, power output during the first half of the time trial was increased, and then decreased during the second half, compared with placebo. The authors attributed this to attenuated afferent feedback from exercising muscles, which is then followed by excessive development of fatigue, and overall deterioration of the ability to ‘dose’ their effort. Besides this report, there are no convincing clinical studies on the effects of narcotics on sports performance, resulting in a lack of evidence for either positive or negative effects on performance, as was also concluded by the authors of a recent review [106].

#### S8: Cannabinoids

Cannabinoids are known to affect perceptual function, and in a review of (non-sport) performance, Huestis [107] concluded that this leads to decreased ability to concentrate and maintain attention. In addition, this review concluded that cannabinoids impair information processing and reaction time, all of which would probably negatively affect sports performance, as concluded in a more recent review [108]. Around the same time, Huestis et al. [109] argued that although there are indications that in some settings cannabis has a detrimental effect on performance, in other settings known effects of cannabis might be beneficial. Examples include sports where vision or muscle relaxation are important, or when anxiety or fear impair the potential of the athlete. However, very few scientific data are available on the effects of cannabinoids on sports performance itself, a conclusion that was also reached in two recent reviews [110, 111]. A double-blind, randomized, placebo-controlled, crossover study has been conducted showing tetrahydrocannabinol (THC) had no effect on handgrip strength and decreased performance in a specific type of submaximal bicycle test compared with placebo in healthy untrained males [112]. This shows there is no evidence for performance enhancement of cannabinoids.

#### S9: Glucocorticoids

Glucocorticoids act on metabolism and the immune system, and, through that mechanism, potentially affect performance. For this reason, systemic doses are prohibited in competition. A recent review showed there are varying results of glucocorticoid treatment in performance tests [113]. The two available controlled studies evaluating maximal exercise test variables failed to show effects on *V*O_2max_ and ventilator threshold of 5 days of dexamethasone in untrained subjects [114], and on *P*_max_ of 4 weeks of budesonide treatment in trained subjects [115]. The effects on short, intense exercise were evaluated in three studies. In untrained men, one-legged knee-extensor exercise time to exhaustion was not affected by 5 days of dexamethasone [116]. In contrast, using a similar dosing scheme, another study found an increase in one-legged knee-extensor exercise time to exhaustion of 29%, and running distance in a certain type of maximal exercise test, namely 20-m shuttle-run test, of 19% [117]. Sprint performance over 30 m was not affected in this study. The authors of the latter study postulated a lack of statistical power in the former study was the cause of this apparent discrepancy in outcomes between the two studies. A third study evaluated the effects of a single dose of prednisone on one-legged hopping, and found an 11% improvement in maximal force of the first bout, but not on subsequent bouts or time to exhaustion in any of the bouts [118]. However, it should be noted that no baseline measurement was performed on the study day and therefore the effects of interoccasion variability cannot be excluded. Similar to these short, intense exercise studies, results from studies investigating types of cycling performance are equivocal. A single dose of 20 mg prednisolone did not affect cycling time to exhaustion in trained males, alone or in combination with 4 mg salbutamol [119], a finding that was confirmed in a similar study [120]. However, a multiple dose of 60 mg prednisolone daily for seven days did increase cycling time to exhaustion in trained males by 28 min (62%), although this performance was not controlled with a baseline measurement [121]. An almost identical study that did include a baseline measurement showed an increase of 91% (50.9 min) in cycling time to exhaustion using the same dosing regimen, combined with intense training in untrained subjects [122]. Although the statistical comparison was made between baseline measurement and post-treatment, and not additionally to the placebo measurements, it seems likely that this is a true effect as there was no change in the placebo treatment. Another study confirmed these findings in untrained females treated with 50 mg prednisone daily for 1 week, which showed a 39% increase (18.5 min) in cycling time to exhaustion [123]. However, it should be noted that it is unclear how time to exhaustion relates to real-life endurance performance, which is usually not until exhaustion but until a finish line is reached. In summary, there is conflicting evidence on the effectiveness of glucocorticoids for improving different performance types. However, there seems to be an effect on specific strength tests and shuttle run time, and multiple, but not single, doses seem to improve time to exhaustion in moderately trained subjects. At the same time, only one study with 10 subjects showed an effect on a relevant performance surrogate marker, namely one-legged hopping maximal force.

### P1: Prohibited in Particular Sports

This category covers substances prohibited in particular sports (i.e. archery, automobile, billiards, darts, golf, shooting, skiing/snowboarding and underwater sports) and contains only the group of β-blockers. This group of substances inhibits β-adrenergic receptors, thereby reducing heart rate, anxiety and tremulousness, which could potentially enhance performance in sports where precision and accuracy are vital. A double-blind, randomized, placebo-controlled, crossover study has been conducted evaluating the effect of metoprolol on shooting performance in amateur marksmen [124]. The study showed that, on average, participants improved their shooting when taking metoprolol compared with placebo, which was especially the case in the more skilled marksmen. It therefore seems that β-blockers do improve shooting performance, and possibly other precision and accuracy sports also included in this category, based on one study of 33 subjects.

## Conclusion

Of all 23 specific substance classes defined in the 2018 Prohibited List, only five classes show evidence of having the ability to enhance actual sports performance (see Table 1). Anabolic agents can increase muscle strength at supratherapeutic doses; β2-agonists can increase muscle strength and peak sprint power at high concentrations; some stimulants increase muscle strength, peak sprint power and decrease 1500 m run time; glucocorticoids can improve muscle strength; and β-blockers can improve accuracy. In addition, for another class there is evidence of performance enhancement, but only in untrained subjects: GH can improve sprint performance. Importantly, there is no robust evidence that any of the substance classes on the Prohibited List have the ability to improve endurance performance. Glucocorticoids improve time to exhaustion, but it is unclear whether this relates to actual endurance performance. Erythropoietin receptor agonists improve *V*O_2max_ and *P*_max_, but the available evidence shows no effect on actual endurance performance. What also becomes clear from this overview is that for most substance classes (11 of 23), there are no well-designed studies evaluating the effects on performance (in trained subjects), meaning there is an absence of level 1 evidence. Physicians involved in administering such substances in particular are performing practices similar to off-license prescribing, and prescribing without evidence is considered bad medical practice. In contrast, for another six substance classes, well-designed studies are available that show some evidence of absence of (relevant) effects on performance. Overall, this review therefore shows that for the majority of substance classes (17 of 23), there is no convincing evidence that they enhance the performance of athletes. Moreover, in regard to the other five classes that are prohibited in all sports that do have evidence-based effects, it is unproven whether such effects are relevant or useful in many types of sport (e.g. endurance sports) as they only improve specific performance tasks (mainly strength and sprint power). Although these aspects do play some role in many sport disciplines (such as, for example, athletics or soccer), it is not very clear whether these effects would also impact actual performance in those disciplines. In any case, there are no studies investigating this, as we have shown. These findings together seem discordant with the general perception that substances on the Prohibited List, by definition, improve performance (to a great extent). This is especially evident when it is considered that a total of only 266 subjects (from 11 studies) form the clinical pharmacological level 1 evidence base for performance enhancement, the main reason for anti-doping efforts. Although the WADA Code only requires evidence for *the potential* to enhance performance, and there are two other criteria that can be applied to make a substance prohibited, we conclude there is a lack of high-level evidence for improvement of actual performance based on this review. Undertaking more high-quality clinical research to provide the level 1 evidence base for the current Prohibited List could fill some of these gaps. Some of this research could be impossible due to practical or ethical objections, but the current level of randomized evidence is low and there appear to be many areas where such research is possible. Furthermore, if there is clear evidence that there are no performance-enhancing effects of a certain class, athletes should be informed of this. This could potentially lead to fewer athletes being tempted to use these substances. Finally, such steps would lead to a more transparent and high-level evidence-based fight against doping, and possibly reduce the efforts and resources needed to test for abuse.

**Table 1:** Overview of all substance classes and evidence for performance enhancement

Substance class	Well-designed studies?	Studies with trained athletes?	Relevant performance parameters showing improvement	No. of trained athletes in studies with relevant performance parameters
S0: Non-approved substances	No	NA	NA	NA
S1: Anabolic agents	Yes	Yes	Muscle strength when combined with strength training + 10–20% bench-press and squatting power [24] + 12% bench press power [25] + 23% and + 12% elbow flexion and knee extension [26]	Total: 91 40 [24] 21 [25] 30 [26]
S2: Peptide hormones, growth factors, related substances and mimetics				
Erythropoietin-receptor agonists	Yes	Yes	No evidence for effects on relevant endurance parameters, only on *V*O_2max_, maximal power output and time to exhaustion	Total: 161 20 [32] 11 [34] 27 [35] 16 [36] 40 [37] 47 [39]
Hypoxia-inducible factor activating agents	No	NA	NA	NA
GATA inhibitors	No	NA	NA	NA
TGFβ inhibitors	No	NA	NA	NA
Innate repair receptor agonists	No	NA	NA	NA
Chorionic gonadotrophin and luteinizing hormones and their releasing factors	No	NA	NA	NA
Corticotrophins and their releasing factors	Yes	Yes	No	Total: 24 16 [54] 8 [55]
Growth hormone, its fragments and releasing factors	Yes	No	Sprint performance + 3.9% Wingate sprint capacity [62] in untrained subjects	Total: 123 22 [59] 30 [60] 7 [61] 64 untrained [62]
Growth factors and growth factor modulators	Yes	No	NA	NA
S3: β2-Agonists	Yes	Yes	Peak sprint power, muscle strength + 6% maximal voluntary isometric contraction, + 11% peak twitch force [70] + 8.4% quadriceps muscle strength, + 2.2% and + 3.3% Wingate peak and mean power, + 3% Wingate total work [71] + 4% (single dose) and + 6% (multiple dose) Wingate peak power [72]	Total: 39 10 [70] 9 [71] 20 [72]
S4: Hormone and metabolic modulators				
Aromatase inhibitors	No	NA	NA	NA
Selective estrogen receptor modulators	No	NA	NA	NA
Other anti-estrogenic substances	No	NA	NA	NA
Agents modifying myostatin function(s)	No	NA	NA	NA
Metabolic modulators	No	NA	NA	NA
S5: Diuretics and masking agents	Yes	Yes	No	Total: 70 62 [89] 8 [90]
S6: Stimulants	Yes	Yes	Muscle strength, sprint performance, 1500 m run time + 9% knee extension strength, + 3% peak Wingate sprint performance [101] 2% decrease 1500 m run time [104]	Total: 58 9 [97] 22 [101] 9 [102] 11 [103] 7 [104]
S7: Narcotics	Yes	Yes	No	Total: 8 8 [105]
S8: Cannabinoids	Yes	No	NA	NA
S9: Glucocorticoids	Yes	Yes	One-legged hopping force + 11% one-legged hopping maximal force [118]	Total: 10 10 [118]
P1: β-Blockers (prohibited in particular sports)	Yes	Yes	Shooting performance + 13.4% shooting performance [124]	Total: 33 33 [124]

*NA* not applicable, *TGF* transforming growth factor, *VO*_*2*_*max* maximal oxygen consumption
